# Pepitope facilitates TCR-neoantigen screen analysis in the R language

**DOI:** 10.1093/bioinformatics/btag542

**Published:** 2026-07-21

**Authors:** Moritz Broft, Wouter Scheper, Michael Schubert

**Affiliations:** Institute of Bioinformatics, Medical University of Innsbruck, Innrain 80, 6020 Innsbruck, Austria; Division of Molecular Oncology and Immunology, Oncode Institute, The Netherlands Cancer Institute, Plesmanlaan 121, Amsterdam 1066 CX, the Netherlands; Institute of Bioinformatics, Medical University of Innsbruck, Innrain 80, 6020 Innsbruck, Austria; Division of Molecular Oncology and Immunology, Oncode Institute, The Netherlands Cancer Institute, Plesmanlaan 121, Amsterdam 1066 CX, the Netherlands

## Abstract

**Motivation:**

Functional screening of patient-derived T cell receptor (TCR)-neoantigen pairs via co-culture experiments is a way to design personalised immunotherapy or to investigate its mechanism of action. Current computational toolkits can either generate and prioritise candidate epitopes from tumour variants or count barcodes in sequencing data. However, they lack modules to support experimental screening, such as sample demultiplexing, construct quality control, and downstream analysis. To bridge these gaps, we present *pepitope*, an R package that integrates minigene library generation, sequencing-based quality control (QC), and differential abundance analysis of co-culture screens into a single software package within the accessible R/Bioconductor ecosystem.

**Results:**

*pepitope* workflows include the extraction of mutant and reference peptides with customisable flanking regions from tumour variant calls using Bioconductor annotation resources; demultiplexing and barcode counting for construct QC; and negative-binomial-based differential testing built on *DESeq2* to identify immunogenic epitopes in TCR co-culture assays. By remaining within R, *pepitope* lowers the barrier for lab-based biologists familiar with R and Bioconductor to perform end-to-end co-culture screen analyses without needing dedicated computational support.

**Availability:**

*pepitope* (R ≥ 4.5.0) is freely available on GitHub under the GPL-3.0 license, with detailed vignettes hosted at https://mschubert.github.io/pepitope/. Installation is facilitated via the *remotes* package in R.

## 1 Introduction

Identification of patient-specific T cell receptor (TCR)-neoantigen interactions via co-culture screening is a pivotal step for advancing personalised cancer immunotherapies. Genetic neoantigen screening platforms such as *HANSolo* [human leukocyte antigen (HLA)-agnostic neoantigen screening)] (Cattaneo *et al.* 2023) utilise libraries of minigenes expressed in autologous B cell lines to unbiasedly interrogate both CD4^+^ and CD8^+^ T cell repertoires across complete patient HLA genotypes. The *amplicon-nf* pipeline, developed in tandem with *HANSolo*, automates barcode counting of sequencing reads from HLA-unbiased screens but requires external peptide design, sample demultiplexing, downstream quality control, and differential abundance testing. Complementarily, tools such as *pVACtools* (Hundal *et al.* 2020) and *Vaxrank* (Rubinsteyn *et al.* 2017) provide comprehensive suites for neoantigen candidate generation, including MHC binding prediction and epitope ranking, but do not include any screen analysis steps. Hence, none of the available tools address the entire workflow from peptide extraction to construct quality control and the statistical analysis of co-culture screening data.

Laboratory-focused researchers often rely on the R/Bioconductor programming environment for statistical analysis and data visualisation, benefitting from extensive documentation, vignettes, and integrated annotation resources. Here, the absence of an R-native solution that spans the entire workflow from peptide construct generation through QC to differential screen analysis creates a barrier to adoption and reproducibility. To fill this gap, we developed *pepitope*, an R package that seamlessly integrates all critical steps of peptide-TCR co-culture screens into a single, user-friendly R/Bioconductor interface, with extensive documentation and usage guides.

## 2 Implementation

**Figure 1:**
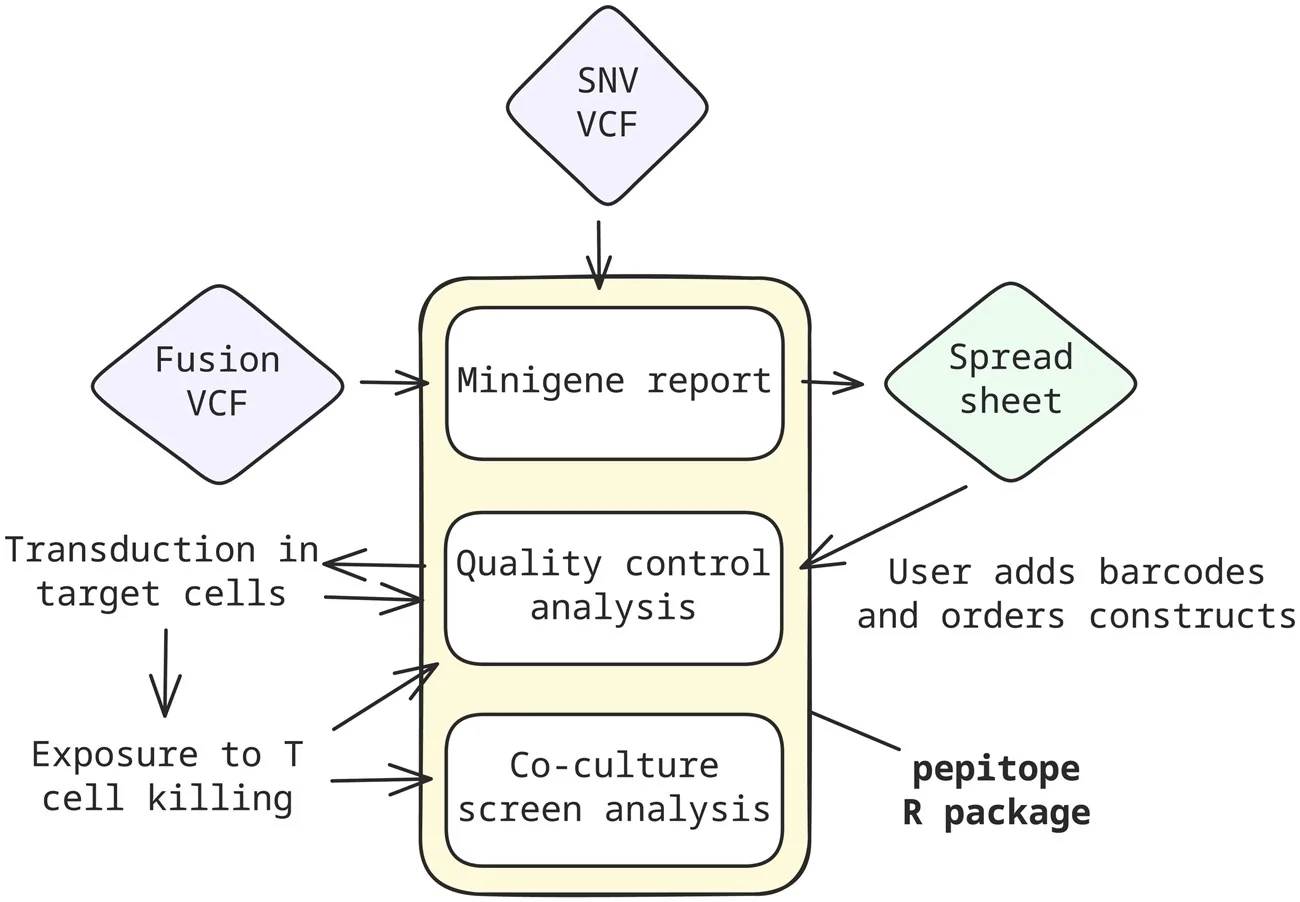
Overview of the *pepitope* workflow. Starting from patient-specific VCF files for small variants from exome sequencing and gene fusions from ribonucleic acid (RNA) data, *pepitope* constructs a minigene table of patient tumour variants, including their peptide context. This table can be used to order synthetic DNA constructs for neoantigen-TCR co-culture screens. *pepitope* can then be used for quality control of these libraries and their transductions, and quantify abundance changes that reflect T cell killing of target cells expressing these neoantigens.

The *pepitope* implementation unifies three essential workflows into a cohesive R/Bioconductor pipeline (Fig. 1): variant-based peptide table creation, construct quality assessment, and co-culture screen analysis. Starting with standard variant call format (VCF) inputs compatible with popular nf-core workflows, users can generate context-aware peptide libraries, ensure consistent barcode representation across multiplexed sequencing runs, and perform differential abundance testing of peptide-TCR interactions, all within the R environment. We demonstrate the latter two workflows on previously published experimental screening data produced from the *HANSolo* platform (Supplementary Data).

### 2.1 Peptide table generation

Users begin with VCF files of tumour variants and optionally gene fusions, typically produced by nf-core pipelines (Ewels *et al.* 2020) such as *sarek*, *rnaseq*, and *rnafusion*, which are widely adopted in sequencing data analysis and core facilities. Within R, *pepitope* annotates coding mutations and fusions against a consistent reference genome and annotation set, producing a peptide table containing both reference and alternative sequences for each event. For single-nucleotide variants, users define a flanking ‘context’ window (default: 15 codons upstream and downstream). Insertions and deletions are captured by spanning the altered region plus flanking codons, and frameshifts include upstream codons plus downstream sequence to the first stop codon. Optionally, known restriction sites in these minigenes can be removed without changing the peptide sequence. Finally, one or more synthetic DNA barcodes are appended to each peptide construct to enable multiplexed tracking in downstream assays. This last step can be carried out manually by the user in a familiar spreadsheet environment.

### 2.2 Quality control of construct libraries

Ensuring uniform representation of each peptide barcode, and hence its construct, is required for accurate quantification of abundance changes. *pepitope* supports the use of published barcode sets from established sources to annotate constructs. In a small-scale QC sequencing run, multiple samples are uniquely tagged via PCR-added sample barcodes, then pooled and sequenced into a single FASTQ file. *pepitope* counts construct-level barcodes for all multiplexed samples, and generates a standardised Bioconductor-structured dataset (*SummarizedExperiment* object) for differential abundance analysis. Diagnostic plots display total read counts per sample and the distribution of barcode abundances (ordering barcodes from least to most frequent) to reveal over- and under-represented constructs, barcode dropout, and cross-sample contamination. Examples of how these look can be found in the package vignette.

### 2.3 Differential abundance analysis of co-culture screens

For functional screening, cells expressing tumour variant-encoding minigenes are co-cultured with T cells of interest alongside experimental controls. Changes in barcode counts reflect TCR-mediated depletion or enrichment of those cells (Cattaneo *et al.* 2023). *pepitope* compares barcode abundances across conditions using the DESeq2 (Love *et al.* 2014) package, yielding fold-change estimates and false discovery rate (FDR)-adjusted *P* values. Results are visualised as MA-plots (mean abundance versus log_2_ fold change) with optional linking of matched reference and mutant peptide constructs to highlight significantly reactive epitopes.

## 3 Conclusion

*pepitope* enables scientists with minimal computational knowledge to perform quality control and screening analyses for TCR-peptide co-culture experiments. It does so by providing the entire workflow in an R package rather than as several disjoint tools.

## Supplementary Material

btag542_Supplementary_Data

## Data Availability

The package and vignettes are accessible via https://mschubert.github.io/pepitope/. Data analyzed in the Supplementary Materials are available in the NCBI Sequence Read Archive with accession number SRR21707791.
