# Supplementary material for: Pepitope facilitates TCR-neoantigen screen analysis in the R language
Source: Bioinformatics. 2026 Jul 21;42(8):btag542. doi: 10.1093/bioinformatics/btag542 (PMC13452055; doi:10.1093/bioinformatics/btag542)
Supplement: Supplementary file 1 [file btag542_supplementary_data.pdf]

Supplementary Data for 'pepitope facilitates TCR-neoantigen screen analysis in the R language'

Reproducing Fig. 1d of Cattaneo et al. (2022)

This script processes the Lib1 CD4 sequencing data from the shared TAA minigene screen described by Cattaneo et al. (https://www.nature.com/articles/s41587-022-01547-0). In this screen, pooled minigene constructs are represented by construct barcodes, transduced into B-cell antigen-presenting cells, and exposed to engineered T cells. Constructs encoding recognized antigens are expected to be depleted after T-cell co-culture, so the downstream analysis treats reduced construct abundance as the screen signal.

The workflow mirrors the `pepitope` vignettes: define sample barcodes, define a construct barcode library, count matching reads into a `SummarizedExperiment`, inspect sample and construct barcode recovery, and use DESeq2-based contrasts to identify depleted constructs. The focused comparison here reproduces the CD4 TCL model-antigen screen shown in Fig. 1d of the paper for the MANSC1 and SNORD73 conditions over the TCL mock controls. It does not process the Lib2 CD8 source file used for the corresponding CD8/DMF4/DMF5 benchmark panels.

```
library(pepitope)
library(dplyr)
library(ggplot2)
```

Input files

The public SRA run is available from the NCBI Trace Archive at https://trace.ncbi.nlm.nih.gov/Traces/?run=SRR21707791. That run lists the CD4 and CD8 source FASTQ files; this script uses the original-format Lib1 CD4 source file listed there, rather than reconstructing the run with `fasterq-dump`. The file `41587_2022_1547_MOESM3_ESM.xlsx` is the paper supplement "Supplementary Table 1".

```
fastq_file = "6782_1_Lib1_CD4_NoIndex_S1_R1_001.fastq.gz"
supp_file = "41587_2022_1547_MOESM3_ESM.xlsx"
```

Sample sheet

The sample sheet follows the `pepitope` convention used in the package vignettes: `barcode` is the sample barcode at the beginning of each read, `origin` is the experimental condition, `rep` is the replicate identifier, and `patient` selects the construct library from `all_constructs`. Here the patient label is `TCL`, matching the T-cell line screen and the construct table below.

The sheet is restricted to the Lib1 CD4 TCL samples needed for the model-antigen screen: MANSC1 and SNORD73 co-cultures, mock controls, barcode-only B-cell controls, and the dilution samples present in the metadata.

```
sample_sheet = tibble::tribble(
    ~sample_id,          ~patient, ~rep, ~origin,  ~barcode,
    "TCL_MANSC1_01",     "TCL",    "01", "MANSC1", "CAGAGTT",
    "TCL_SNORD73_01",    "TCL",    "01", "SNORD73","GCACGAA",
    "TCL_1_1_8_01",      "TCL",    "01", "D8",     "GCGAACT",
    "TCL_1_1_98_01",     "TCL",    "01", "D98",    "GCTTCCT",
    "TCL_1_1_331_01",    "TCL",    "01", "D331",   "TGCCAAT",
```

```
    "TCL_1_1_998_01",   "TCL",    "01", "D998",   "TGGAAGC",
    "TCL_MANSC1_02",    "TCL",    "02", "MANSC1", "TGTTGGT",
    "TCL_SNORD73_02",   "TCL",    "02", "SNORD73","CAGTCAT",
    "TCL_1_1_8_02",     "TCL",    "02", "D8",     "CATTCTG",
    "TCL_1_1_98_02",    "TCL",    "02", "D98",    "TAGCTAG",
    "TCL_1_1_331_02",   "TCL",    "02", "D331",   "CGTCCTT",
    "TCL_1_1_998_02",   "TCL",    "02", "D998",   "AATTGCC",
    "TCL_mock_01",      "TCL",    "01", "Mock",   "GTTGCCA",
    "TCL_mock_02",      "TCL",    "02", "Mock",   "CTCATCT",
    "TCL_Bc_only_01",   "TCL",    "01", "Bcell",  "CGCAATT",
    "TCL_Bc_only_02",   "TCL",    "02", "Bcell",  "TTCGAAC"
)
```

Construct library

The shared TAA minigene library is listed in the sheet "Supp. Table 1B.Minigene library" of `41587_2022_1547_MOESM3_ESM.xlsx`, the paper supplement "Supplementary Table 1". Each row describes one minigene oligo and its displayed peptide sequence. The construct barcodes are not stored in that sheet, so this script uses the barcode list referenced by the `pepitope` vignette and assigns the first 4,764 barcodes by `OLIGO NR`, matching the size and order of the Table 1B library.

Some entries occur as matched mutant and wild-type peptide pairs, encoded in `GENE NAME` with `mut` and `wt` suffixes. For those paired entries, the shared base name is used as both `gene_name` and `mut_id`, while `pep_type` is set to `alt` for mutant peptides and `ref` for wild-type peptides. This gives `plot_screen()` the same ref/alt structure used by the pepitope examples.

```
url = "https://raw.githubusercontent.com/hawkjo/freebarcodes/master/barcodes/barcodes12-1.txt"
valid_barcodes = readr::read_tsv(url, col_names=FALSE, show_col_types=FALSE)$X1

constructs = readxl::read_xlsx(supp_file, sheet="Supp. Table 1B.Minigene library") |>
    tidyr::fill(`GENE NAME`) |>
    mutate(
        raw_gene_name = `GENE NAME`,
        gene_base = sub("(mut|wt)$", "", raw_gene_name),
        gene_suffix = case_when(
            grepl("mut$", raw_gene_name) ~ "mut",
            grepl("wt$", raw_gene_name) ~ "wt",
            TRUE ~ NA_character_
        )
    ) |>
    group_by(gene_base) |>
    mutate(
        has_pair = all(c("mut", "wt") %in% gene_suffix)
    ) |>
    ungroup() |>
    transmute(
        gene_name = ifelse(has_pair, gene_base, raw_gene_name),
        mut_id = ifelse(has_pair, gene_base, raw_gene_name),
        pep_id = raw_gene_name,
        pep_type = case_when(
            has_pair & grepl("mut$", raw_gene_name) ~ "alt",
            has_pair & grepl("wt$", raw_gene_name) ~ "ref",
            TRUE ~ NA_character_
        ),
```

```r
        tiled = `MINIGENE SEQ`,
        barcode = valid_barcodes[`OLIGO NR`]
    )

all_constructs = list(TCL=constructs)

constructs
#> # A tibble: 4,764 x 6
#>    gene_name mut_id pep_id pep_type
#>    <chr>     <chr>  <chr>  <chr>
#>  1 MAGEA1    MAGEA1 MAGEA1 <NA>
#>  2 MAGEA1    MAGEA1 MAGEA1 <NA>
#>  3 MAGEA1    MAGEA1 MAGEA1 <NA>
#>  4 MAGEA1    MAGEA1 MAGEA1 <NA>
#>  5 MAGEA1    MAGEA1 MAGEA1 <NA>
#>  6 MAGEA1    MAGEA1 MAGEA1 <NA>
#>  7 MAGEA1    MAGEA1 MAGEA1 <NA>
#>  8 MAGEA1    MAGEA1 MAGEA1 <NA>
#>  9 MAGEA1    MAGEA1 MAGEA1 <NA>
#> 10 MAGEA1    MAGEA1 MAGEA1 <NA>
#>    tiled                                                                    barcode
#>    <chr>                                                                    <chr>
#>  1 ATGAGCCTGGAACAGAGAAGCCTGCACTGCAAGCCTGAGGAAGCCCTGGAAGCTCAGCAAGAGGCTCT~ AACAAC~
#>  2 GAAGCTCAGCAAGAGGCTCTGGGACTTGTGTGTGTGCAGGCCGCCACATCTAGCTCTAGTCCTCTGGT~ AACAAC~
#>  3 TCTAGCTCTAGTCCTCTGGTGCTGGGCACCCTGGAAGAAGTGCCTACAGCCGGCTCTACCGATCCTCC~ AACAAC~
#>  4 GCCGGCTCTACCGATCCTCCTCAATCTCCTCAAGGCGCCAGCGCCTTTCCTACCACCATCAACTTCAC~ AACAAC~
#>  5 CCTACCACCATCAACTTCACCCGGCAGAGACAGCCTAGCGAGGGCAGCAGCTCTAGAGAGGAAGAGGG~ AACAAC~
#>  6 AGCTCTAGAGAGGAAGAGGGCCCTAGCACCAGCTGCATCCTGGAAAGCCTGTTCAGAGCCGTGATCAC~ AACAAC~
#>  7 CTGTTCAGAGCCGTGATCACCAAGAAAGTGGCCGACCTCGTGGGCTTCCTGCTGCTGAAGTACAGAGC~ AACAAC~
#>  8 CTGCTGCTGAAGTACAGAGCCAGAGAACCCGTGACCAAGGCCGAGATGCTGGAAAGCGTGATCAAGAA~ AACAAC~
#>  9 CTGGAAAGCGTGATCAAGAACTACAAGCACTGCTTCCCCGAGATCTTCGGCAAGGCCTCTGAGTCTCT~ AACAAC~
#> 10 GGCAAGGCCTCTGAGTCTCTGCAGCTCGTGTTCGGCATCGACGTGAAAGAGGCCGATCCTACCGGCCA~ AACAAC~
#> # i 4,754 more rows
```

Read structure and counting

`pepitope` can infer the read structure from the sample and construct barcode matches in the reads. This keeps the script tied to the barcode definitions above instead of hard-coding offsets in the Rmd. The diagnostic plot should show the sample barcode at the start of the read and the construct barcode at the position where the library barcode is recovered.

```r
plot_read_structure(fastq_file, sample_sheet, all_constructs)
```

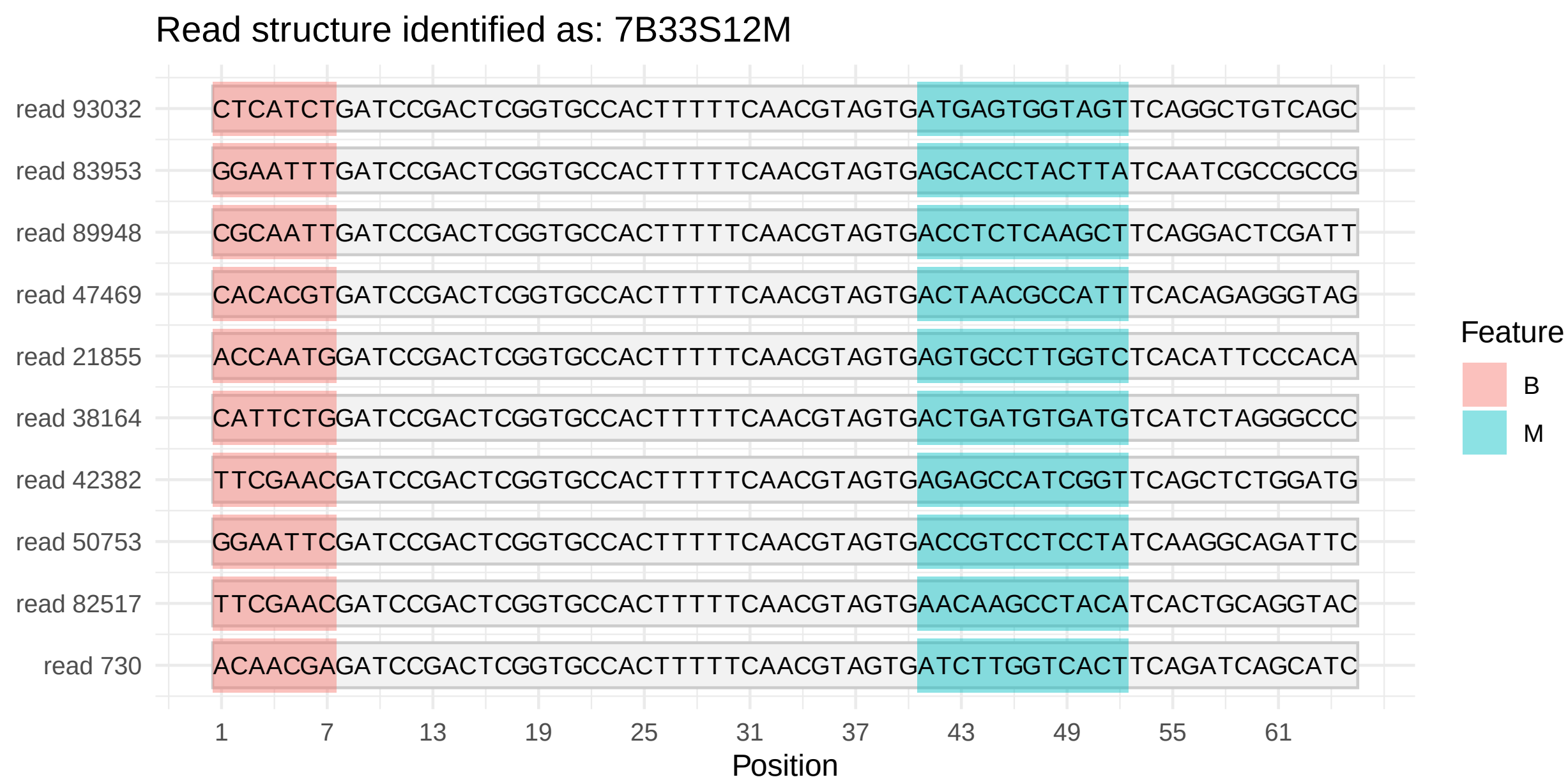

`count_fastq()` counts reads whose sample barcode and construct barcode both match the supplied tables. The result is a `SummarizedExperiment` with construct counts in the assay, sample metadata in `colData()`, and construct annotations in `rowData()`.

```
dset = count_fastq(
    fastq_file,
    sample_sheet,
    all_constructs,
    valid_barcodes
)
#> Read structure identified as '7B33S12M'
#> Counting barcodes in 6782_1_Lib1_CD4_NoIndex_S1_R1_001.fastq.gz
#> Processed 503,897,446 reads
```

We can inspect the resulting object including `colData` for the sample metadata and `rowData` for the construct annotations. The assay contains the read counts for each construct in each sample, which are used for the downstream DESeq2 contrasts.

```
dset
#> class: SummarizedExperiment
#> dim: 17214 16
#> metadata(0):
#> assays(1): counts
#> rownames(17214): AACAACAACACC AACAACAACGGT ... TTGTTGGAATGG
#>   TTGTTGTGGTAC
#> rowData names(7): barcode bc_type ... pep_type tiled
#> colnames(16): TCL_MANSC1_01 TCL_SNORD73_01 ... TCL_Bc_only_01
#>   TCL_Bc_only_02
#> colData names(10): sample_id patient ... short label
colData(dset)
#> DataFrame with 16 rows and 10 columns
#>                     sample_id  patient         rep      origin     barcode
#>                   <character> <factor> <character> <character> <character>
#> TCL_MANSC1_01    TCL_MANSC1_01      TCL          01      MANSC1     CAGAGTT
#> TCL_SNORD73_01 TCL_SNORD73_01      TCL          01     SNORD73     GCACGAA
```

```
#> TCL_1_1_8_01     TCL_1_1_8_01      TCL          01          D8      GCGAACT
#> TCL_1_1_98_01   TCL_1_1_98_01      TCL          01         D98      GCTTCCT
#> TCL_1_1_331_01 TCL_1_1_331_01      TCL          01        D331      TGCCAAT
#> ...                       ...      ...         ...         ...          ...
#> TCL_1_1_998_02 TCL_1_1_998_02      TCL          02        D998      AATTGCC
#> TCL_mock_01       TCL_mock_01      TCL          01        Mock      GTTGCCA
#> TCL_mock_02       TCL_mock_02      TCL          02        Mock      CTCATCT
#> TCL_Bc_only_01 TCL_Bc_only_01      TCL          01       Bcell      CGCAATT
#> TCL_Bc_only_02 TCL_Bc_only_02      TCL          02       Bcell      TTCGAAC
#>                total_reads mapped_reads         smp          short
#>                  <numeric>    <numeric> <character>    <character>
#> TCL_MANSC1_01     13750924     12966793   MANSC1-01  TCL MANSC1-01
#> TCL_SNORD73_01    13069333     12440838  SNORD73-01 TCL SNORD73-01
#> TCL_1_1_8_01      15055957     14249997       D8-01      TCL D8-01
#> TCL_1_1_98_01     16143904     15062283      D98-01     TCL D98-01
#> TCL_1_1_331_01    14834540     13946115     D331-01    TCL D331-01
#> ...                    ...          ...         ...            ...
#> TCL_1_1_998_02    14363266     13422962     D998-02    TCL D998-02
#> TCL_mock_01       14556874     13667331     Mock-01    TCL Mock-01
#> TCL_mock_02       16034640     14854986     Mock-02    TCL Mock-02
#> TCL_Bc_only_01    36916863     34067380    Bcell-01   TCL Bcell-01
#> TCL_Bc_only_02    32685164     30331260    Bcell-02   TCL Bcell-02
#>                                  label
#>                            <character>
#> TCL_MANSC1_01  TCL MANSC1-01 (TCL_M..
#> TCL_SNORD73_01 TCL SNORD73-01 (TCL_..
#> TCL_1_1_8_01   TCL D8-01 (TCL_1_1_8..
#> TCL_1_1_98_01  TCL D98-01 (TCL_1_1_..
#> TCL_1_1_331_01 TCL D331-01 (TCL_1_1..
#> ...                                ...
#> TCL_1_1_998_02 TCL D998-02 (TCL_1_1..
#> TCL_mock_01    TCL Mock-01 (TCL_moc..
#> TCL_mock_02    TCL Mock-02 (TCL_moc..
#> TCL_Bc_only_01 TCL Bcell-01 (TCL_Bc..
#> TCL_Bc_only_02 TCL Bcell-02 (TCL_Bc..
```

Quality control

The read-count plot summarizes how many reads are assigned to each sample after barcode matching. The barcode-distribution plot checks whether the construct library is broadly represented or dominated by a small number of constructs. As in the pepitope vignette, these plots are inspected before interpreting the DESeq2 contrasts.

```
plot_read_count(dset)
```

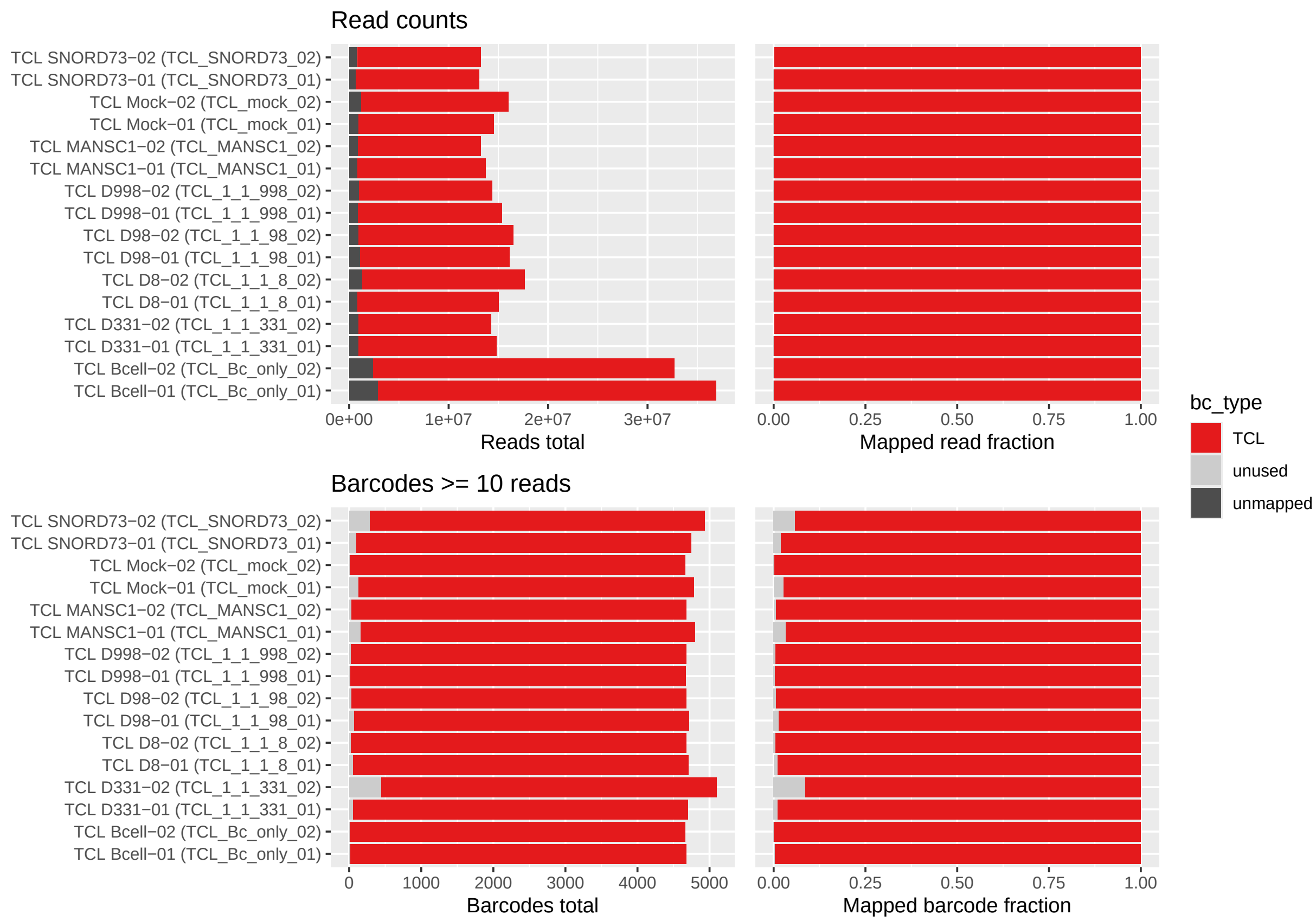

In the read counts, we see that every sample is well represented and and only few are unmapped (not present in the list of valid barcodes) or unused (present in the list but not assigned in the construct annotations).

The barcode distribution plot also shows that the library is broadly represented, with most constructs having a similar amount of reads within all samples. The number of constructs with zero reads is small, and the distribution does not appear dominated by a small number of constructs with very high read counts. This suggests that the screen is well represented and that the DESeq2 contrasts will be based on a good sampling of the library, rather than a few highly abundant constructs.

```
plot_read_distr(dset)
```

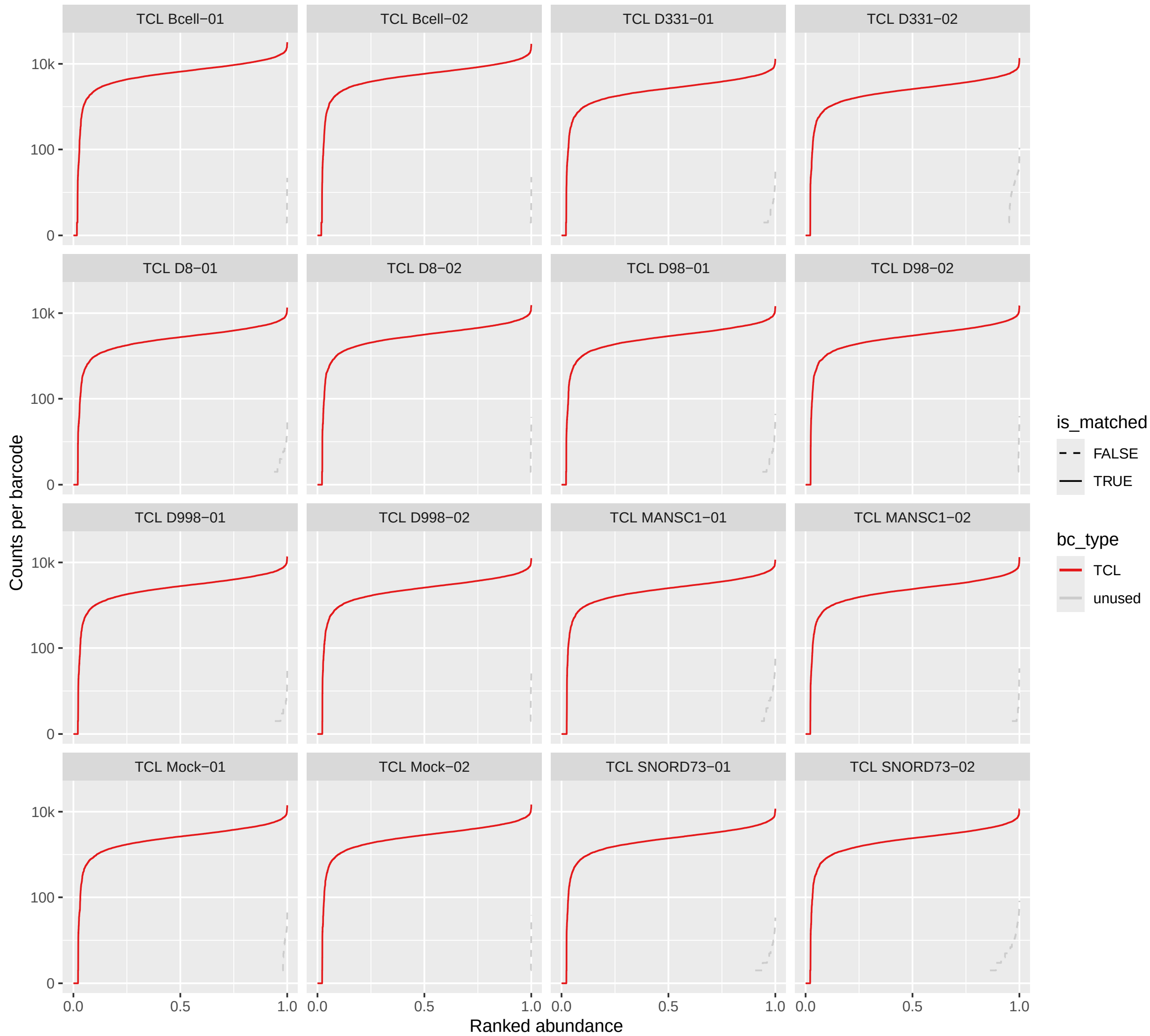

MANSC1 over mock

This contrast compares MANSC1 co-culture samples against TCL mock controls. In the MA plot, the x-axis reflects mean normalized abundance and the y-axis shows log2 fold change for MANSC1 over Mock. Depleted constructs therefore appear with negative log2 fold change, consistent with recognition and loss of the corresponding antigen-presenting cells.

```
dset_mansc1 = dset[, dset$origin %in% c("MANSC1", "Mock")]

res_mansc1 = screen_calc(dset_mansc1, c("MANSC1", "Mock"))
#> converting counts to integer mode
#> Warning in DESeq2::DESeqDataSet(dset, ~rep + origin): some variables in design
#> formula are characters, converting to factors
#> using pre-existing size factors
#> estimating dispersions
#> gene-wise dispersion estimates
#> mean-dispersion relationship
#> final dispersion estimates
```

```
#> fitting model and testing

res_mansc1 |> arrange(padj, pvalue)
#> # A tibble: 6,148 x 13
#>    barcode      baseMean log2FoldChange  lfcSE   stat   pvalue     padj bc_type
#>    <chr>           <dbl>          <dbl>  <dbl>  <dbl>    <dbl>    <dbl> <fct>
#>  1 ACCGCAGTGATA    4401.         -2.11  0.125  -16.9  3.36e-64 2.07e-60 TCL
#>  2 ATAGCCTGTAGC    2703.         -2.18  0.130  -16.8  3.61e-63 1.11e-59 TCL
#>  3 ACCTACCTTACA     753.          2.16  0.260    8.29 1.13e-16 2.31e-13 TCL
#>  4 ATGATAGTACCG    2514.         -0.621 0.130   -4.78 1.75e- 6 2.69e- 3 TCL
#>  5 ACCTCTATAACC    1049.          0.976 0.228    4.29 1.81e- 5 2.00e- 2 TCL
#>  6 AAGATCCGTTGC    3902.         -0.466 0.109   -4.27 1.96e- 5 2.00e- 2 TCL
#>  7 ATCAACCTGCCA    2138.          1.96  0.474    4.14 3.43e- 5 3.02e- 2 TCL
#>  8 ATCAGCGCGGTT    4906.         -0.382 0.0933  -4.09 4.26e- 5 3.27e- 2 TCL
#>  9 ATGCAGCGTGTT     150.          2.16  0.609    3.55 3.84e- 4 2.57e- 1 TCL
#> 10 AGTCCGCCTATG    4551.          0.342 0.0969   3.53 4.18e- 4 2.57e- 1 TCL
#>    gene_name mut_id    pep_id    pep_type tiled
#>    <chr>     <chr>     <chr>     <chr>    <chr>
#>  1 MANSC1    MANSC1    MANSC1mut alt      ACCAAGAACATCAGCGGCGACAAGGCCTGCAACCTGA~
#>  2 MANSC1    MANSC1    MANSC1mut alt      ACCAAGAACATCAGCGGCGACAAGGCCTGCAACCTGA~
#>  3 CMV_pp65  CMV_pp65  CMV_pp65  <NA>     ATCGACCTGCTGCTCCAGAGAGGCCCTCAGTACTCTG~
#>  4 EBV_BZLF1 EBV_BZLF1 EBV_BZLF1 <NA>     GTGTCCGACATCACCCAGAACCAGCAGACAAATCAGG~
#>  5 FLU_PB1   FLU_PB1   FLU_PB1   <NA>     GATGGCGGCCCTAACCTGTACAACATCCGGAACCTGC~
#>  6 SAGE1     SAGE1     SAGE1     <NA>     AAGCCCCAGACCGACAAAGTGATCAGCAACGACGCTC~
#>  7 CMV_pp65  CMV_pp65  CMV_pp65  <NA>     ATCGACCTGCTGCTCCAGAGAGGCCCTCAGTACTCTG~
#>  8 FLU_PB1   FLU_PB1   FLU_PB1   <NA>     AAGAGCTACATCAACCGGACCGGGACCTTCGAGTTCA~
#>  9 EBV_EBNA1 EBV_EBNA1 EBV_EBNA1 <NA>     GGTGCTGGCGGAGCTGGTGCTGGTGGTGCAGGCGGAG~
#> 10 MUC4      MUC4      MUC4      <NA>     CCTCCAGCCTTTACCGACAGCAGATGCTTTCTGGCCG~
#> # i 6,138 more rows
```

```
plot_screen(res_mansc1, min_reads=100, cap_fc=3) + ggtitle("MANSC1 vs Mock")
#> Registered S3 methods overwritten by 'ggpp':
#>   method                  from
#>   heightDetails.titleGrob ggplot2
#>   widthDetails.titleGrob  ggplot2
#> Warning: Removed 1532 rows containing missing values or values outside the
#> scale range (`geom_point()`).
```

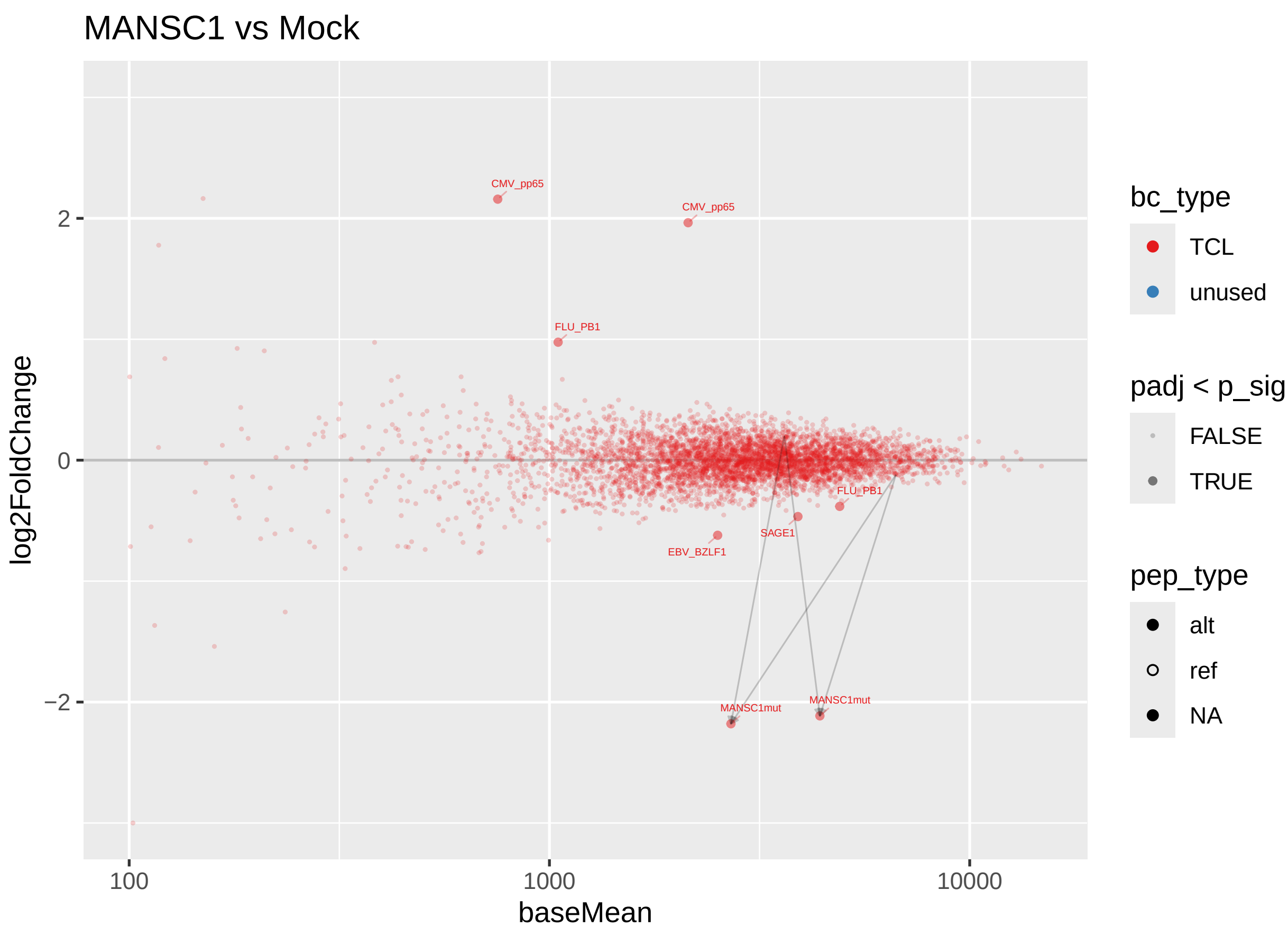

SNORD73 over mock

This contrast repeats the same analysis for SNORD73 over the TCL mock controls.

```
dset_snord73 = dset[, dset$origin %in% c("SNORD73", "Mock")]

res_snord73 = screen_calc(dset_snord73, c("SNORD73", "Mock"))
#> converting counts to integer mode
#> Warning in DESeq2::DESeqDataSet(dset, ~rep + origin): some variables in design
#> formula are characters, converting to factors
#> using pre-existing size factors
#> estimating dispersions
#> gene-wise dispersion estimates
#> mean-dispersion relationship
#> final dispersion estimates
#> fitting model and testing

res_snord73 |> arrange(padj, pvalue)
#> # A tibble: 7,323 x 13
#>   barcode      baseMean log2FoldChange lfcSE  stat   pvalue     padj bc_type
#>   <chr>           <dbl>          <dbl> <dbl> <dbl>    <dbl>    <dbl> <fct>
#> 1 ACCGATTCTGAG    1389.          -2.80 0.177 -15.8 3.51e-56 2.57e-52 TCL
#> 2 ATAGCCACTTAG    1019.          -3.09 0.207 -14.9 4.66e-50 1.71e-46 TCL
#> 3 ATCAACCTGCCA    1935.           1.79 0.181   9.86 5.91e-23 1.44e-19 TCL
#> 4 ACCTACCTTACA     723.           2.08 0.235   8.85 8.70e-19 1.59e-15 TCL
```

```
#>   5 AGTGATCTGTTC    3722.         -0.439 0.110   -3.99 6.71e- 5 9.83e- 2 TCL
#>   6 AAGTTCGGATTG    4134.         -0.392 0.100   -3.92 8.86e- 5 1.08e- 1 TCL
#>   7 AGAGTGTGCTGC    3519.          0.402 0.107    3.74 1.82e- 4 1.90e- 1 TCL
#>   8 AGAACCTACCGC    7779.          0.278 0.0749   3.71 2.11e- 4 1.93e- 1 TCL
#>   9 AGGAGATTCGTT    2165.         -0.510 0.139   -3.67 2.44e- 4 1.99e- 1 TCL
#> 10 AATCGTACAGAC    5089.         -0.327 0.0919  -3.56 3.69e- 4 2.70e- 1 TCL
#>    gene_name mut_id   pep_id      pep_type tiled
#>    <chr>     <chr>    <chr>       <chr>    <chr>
#>  1 SNORD73A  SNORD73A SNORD73Amut alt      ATCAGAAAGACCAGCTACGCCCAGCACCAGCAAGTG~
#>  2 SNORD73A  SNORD73A SNORD73Amut alt      ATCAGAAAGACCAGCTACGCCCAGCACCAGCAAGTG~
#>  3 CMV_pp65  CMV_pp65 CMV_pp65    <NA>     ATCGACCTGCTGCTCCAGAGAGGCCCTCAGTACTCT~
#>  4 CMV_pp65  CMV_pp65 CMV_pp65    <NA>     ATCGACCTGCTGCTCCAGAGAGGCCCTCAGTACTCT~
#>  5 EPHA2     EPHA2    EPHA2       <NA>     GGCGTGTCCGGACTGGTCACCAGCAGAAGCTTTAGA~
#>  6 TYR       TYR      TYR         <NA>     ATGCTGCTGGCCGTGCTGTACTGTCTGCTGTGGTCC~
#>  7 XAGE4     XAGE4    XAGE4       <NA>     CCTCCTCCTGAACTGATCGGCCCTATGCTGGAACCC~
#>  8 MAGEC1    MAGEC1   MAGEC1      <NA>     CCGCCTGAGGGACCCGCTCAATCCCCTCTCCAATCT~
#>  9 PRAME     PRAME    PRAME       <NA>     TTCAAGGCCGTGCTGGATGGCCTGGATGTGCTGCTG~
#> 10 CEACAM5   CEACAM5  CEACAM5     <NA>     GGCAGAGAGATCATCTACCCCAACGCTTCCCTGCTG~
#> # i 7,313 more rows
```

```
plot_screen(res_snord73, min_reads=100, cap_fc=3) + ggtitle("SNORD73 vs Mock")
#> Warning: Removed 2709 rows containing missing values or values outside the
#> scale range (`geom_point()`).
```

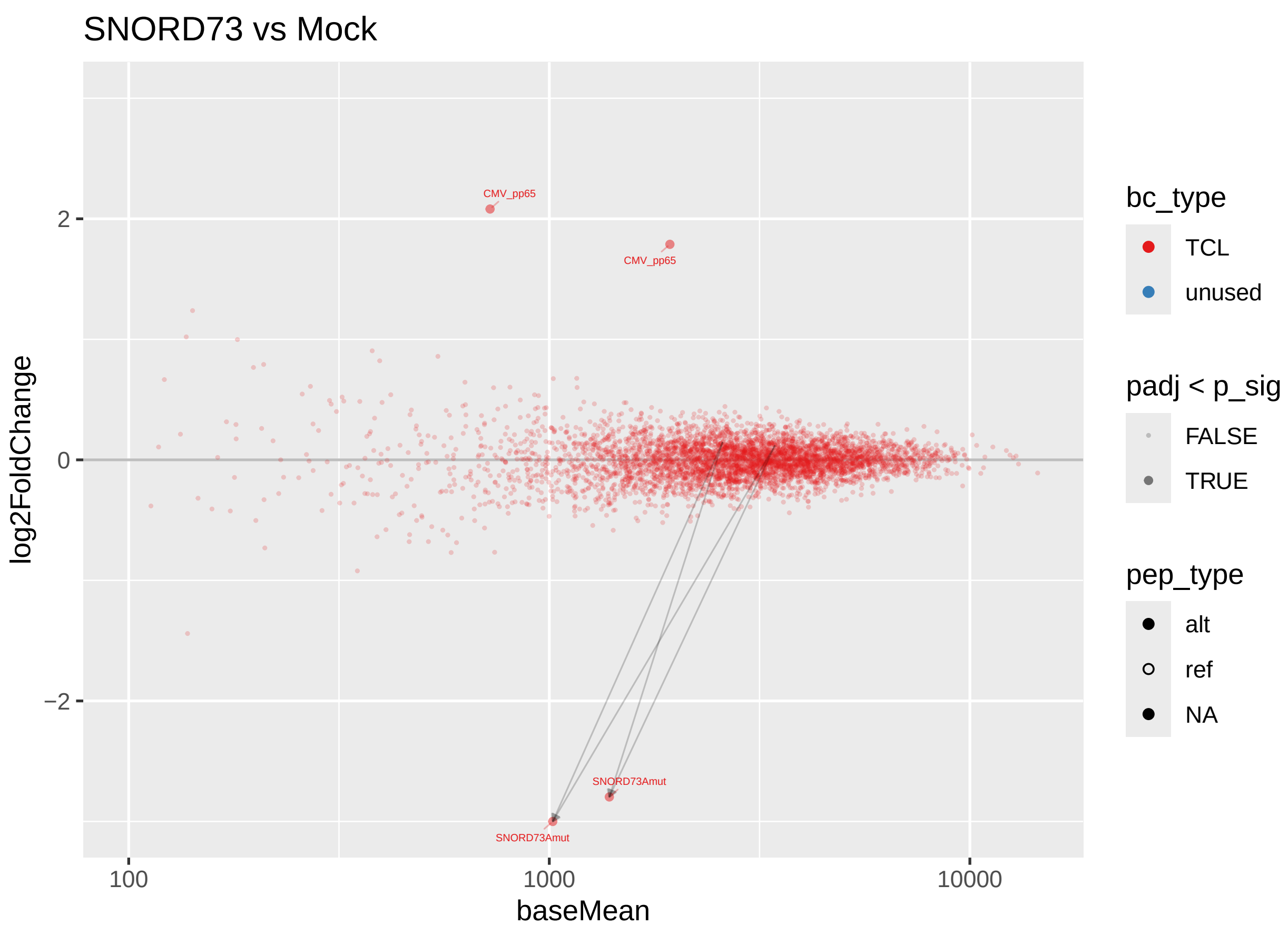

Session info

```r
sessionInfo()
#> R version 4.6.0 (2026-04-24)
#> Platform: x86_64-pc-linux-gnu
#> Running under: Rocky Linux 9.8 (Blue Onyx)
#>
#> Matrix products: default
#> BLAS/LAPACK: FlexiBLAS NETLIB;  LAPACK version 3.12.0
#>
#> locale:
#>  [1] LC_CTYPE=en_US.UTF-8       LC_NUMERIC=C
#>  [3] LC_TIME=en_US.UTF-8        LC_COLLATE=en_US.UTF-8
#>  [5] LC_MONETARY=en_US.UTF-8    LC_MESSAGES=en_US.UTF-8
#>  [7] LC_PAPER=en_US.UTF-8       LC_NAME=C
#>  [9] LC_ADDRESS=C               LC_TELEPHONE=C
#> [11] LC_MEASUREMENT=en_US.UTF-8 LC_IDENTIFICATION=C
#>
#> time zone: Europe/Vienna
#> tzcode source: system (glibc)
#>
#> attached base packages:
#> [1] stats     graphics  grDevices utils     datasets  methods   base
#>
#> other attached packages:
#> [1] ggplot2_4.0.3  dplyr_1.2.1    pepitope_0.4.0
#>
#> loaded via a namespace (and not attached):
#>   [1] DBI_1.3.0                   bitops_1.0-9
#>   [3] polynom_1.4-1               readxl_1.5.0
#>   [5] rlang_1.2.0                 magrittr_2.0.5
#>   [7] otel_0.2.0                  matrixStats_1.5.0
#>   [9] compiler_4.6.0              RSQLite_3.53.1
#>  [11] GenomicFeatures_1.64.0      png_0.1-9
#>  [13] vctrs_0.7.3                 reshape2_1.4.5
#>  [15] stringr_1.6.0               ProtGenerics_1.44.0
#>  [17] pkgconfig_2.0.3             crayon_1.5.3
#>  [19] fastmap_1.2.0               XVector_0.52.0
#>  [21] labeling_0.4.3              utf8_1.2.6
#>  [23] Rsamtools_2.28.0            rmarkdown_2.31
#>  [25] tzdb_0.5.0                  UCSC.utils_1.8.0
#>  [27] purrr_1.2.2                 bit_4.6.0
#>  [29] xfun_0.57                   cachem_1.1.0
#>  [31] cigarillo_1.2.0             GenomeInfoDb_1.48.0
#>  [33] jsonlite_2.0.0              blob_1.3.0
#>  [35] DelayedArray_0.38.2         BiocParallel_1.46.0
#>  [37] parallel_4.6.0              R6_2.6.1
#>  [39] VariantAnnotation_1.58.0    stringi_1.8.7
#>  [41] RColorBrewer_1.1-3          rtracklayer_1.72.0
#>  [43] GenomicRanges_1.64.0        cellranger_1.1.0
#>  [45] Rcpp_1.1.1-1.1              Seqinfo_1.2.0
#>  [47] SummarizedExperiment_1.42.0 knitr_1.51
#>  [49] readr_2.2.0                 IRanges_2.46.0
#>  [51] Matrix_1.7-5                tidyselect_1.2.1
```

```
#>  [53] dichromat_2.0-0.1          abind_1.4-8
#>  [55] yaml_2.3.12                codetools_0.2-20
#>  [57] curl_7.1.0                 lattice_0.22-9
#>  [59] tibble_3.3.1               plyr_1.8.9
#>  [61] Biobase_2.72.0             withr_3.0.2
#>  [63] KEGGREST_1.52.0            S7_0.2.2
#>  [65] evaluate_1.0.5             ggpp_0.6.0
#>  [67] Biostrings_2.80.1          pillar_1.11.1
#>  [69] MatrixGenerics_1.24.0      stats4_4.6.0
#>  [71] generics_0.1.4             vroom_1.7.1
#>  [73] RCurl_1.98-1.18            ensembldb_2.36.1
#>  [75] S4Vectors_0.50.1           hms_1.1.4
#>  [77] scales_1.4.0               glue_1.8.1
#>  [79] lazyeval_0.2.3             tools_4.6.0
#>  [81] BiocIO_1.22.0              locfit_1.5-9.12
#>  [83] BSgenome_1.80.0            GenomicAlignments_1.48.0
#>  [85] XML_3.99-0.23              grid_4.6.0
#>  [87] tidyr_1.3.2                AnnotationDbi_1.74.0
#>  [89] patchwork_1.3.2            restfulr_0.0.16
#>  [91] cli_3.6.6                  S4Arrays_1.12.0
#>  [93] AnnotationFilter_1.36.0    gtable_0.3.6
#>  [95] DESeq2_1.52.0              digest_0.6.39
#>  [97] BiocGenerics_0.58.1        ggrepel_0.9.8
#>  [99] SparseArray_1.12.2         rjson_0.2.23
#> [101] farver_2.1.2               memoise_2.0.1
#> [103] htmltools_0.5.9            lifecycle_1.0.5
#> [105] httr_1.4.8                 MASS_7.3-65
#> [107] bit64_4.8.2
```
